# Editorial: Women in thyroid endocrinology 2022

**DOI:** 10.3389/fendo.2025.1569769

**Published:** 2025-02-25

**Authors:** Laura Sterian Ward, Clara Ugolini, Rossella Elisei

**Affiliations:** ^1^ Laboratory of Cancer Molecular Genetics, School of Medical Sciences, University of Campinas (UNICAMP), Campinas, SP, Brazil; ^2^ Department of Surgical, Medical, Molecular Pathology and Critical Area, Unit of Pathology, University Hospital of Pisa, Pisa, Italy; ^3^ Department of Clinical and Experimental Medicine, Unit of Endocrinology, University Hospital of Pisa, Pisa, Italy

**Keywords:** thyroidology, diversity, gender, science, global contributions, women

It is with great pleasure that we present this Research Topic, highlighting the outstanding work of women researchers in thyroidology. The field of thyroid science has advanced significantly over many decades owing to the contributions of talented female scientists and clinicians. This Research Topic showcases some of the cutting-edge research conducted by women across various aspects of thyroid diseases and biology.

Several papers focus on novel diagnostic approaches for thyroid disorders. Stoian et al. present an innovative multiparametric ultrasound algorithm for evaluating diffuse thyroid disease by combining the B-mode, Doppler, and elastography techniques. This stepwise approach shows promise for improving the diagnostic accuracy and guiding appropriate treatment decisions. Li et al. examine sex-specific associations between pituitary-thyroid hormone axis alterations and thyroid nodules in a large cohort of Chinese adults. Their findings on age-related trends in thyroid parameters and nodule prevalence provide important insights into the potential mechanisms underlying the higher incidence of nodules in women. Lieber et al. conducted a Delphi consensus study on the diagnosis and treatment of subclinical hypothyroidism, revealing diverse attitudes among clinicians and suggesting that current guidelines may need reconsideration. Macvanin et al. provide a comprehensive review of new biomarkers for the diagnosis and monitoring of thyroid disease, discussing the potential of various molecular markers including mRNA, microRNAs, long non-coding RNAs, and circular RNAs.

Regarding thyroid autoimmunity and fertility, Zhang et al. investigated the impact of thyroid autoantibodies on embryo development in women with diminished ovarian reserves undergoing IVF. Their results suggest that thyroid autoimmunity may impair oocyte and embryo quality even in euthyroid women. This highlights the importance of considering thyroid autoimmunity in fertility treatment. Li et al. conducted both a retrospective analysis and meta-analysis on the effects of thyroid autoimmunity in women with unexplained infertility undergoing intrauterine insemination. While their single-center study found lower clinical pregnancy and live birth rates in women with thyroid autoimmunity, the meta-analysis results were not statistically significant, indicating that further research is required in this area.

The relationship between thyroid hormones and pregnancy is discussed by Du et al. who examined the relationship between isolated hypothyroxinemia in early pregnancy and adverse pregnancy outcomes in a prospective cohort study. Interestingly, they found an association only with an increased risk of macrosomia but not with other adverse outcomes. This adds nuance to our understanding of the impact of mild thyroid dysfunction during pregnancy. Liang et al. used Mendelian randomization to investigate the causal relationship between thyroid function and anti-Müllerian hormone level, an important marker of ovarian reserve. Their findings suggest no causal association between genetically predicted thyroid function and anti-Müllerian hormone levels in European populations.

Yuan et al. explore the relationship between bisphenol A exposure and autoimmune thyroid disease in women of childbearing age and found a correlation between urinary bisphenol A and free T4 levels, but no clear association with autoimmune thyroid disease.

Popa et al. investigated the impact of the COVID-19 pandemic on thyroid nodular disease management and found a significant decrease in surgeries, but an increase in the diagnosis of malignant tumors, especially aggressive forms. This highlights the importance of maintaining access to thyroid care even during global health crises.

Di Stefano et al. present an interesting case report of asymptomatic ectopic thyroid tissue discovered incidentally in the liver, highlighting the importance of considering rare presentations of thyroid tissue in differential diagnoses.

These papers collectively demonstrate the diverse and impactful research being conducted by women in thyroidology across clinical, translational, and basic scientific realms. By developing new diagnostic tools to elucidate the mechanisms of thyroid autoimmunity to clarifying the impacts of thyroid dysfunction on fertility and pregnancy, female researchers are at the forefront of advancing our field.

The contribution of women to thyroid science cannot be overstated. Historically, pioneers in thyroid hormone discovery, such as Rosalind Pitt Rivers, who co-discovered triiodothyronine, and Valerie Anne Galton, who explored thyroid hormone metabolism, paved the way for future generations. Today, women continue to lead groundbreaking research, head major thyroid associations, and mentor the next generation of thyroidologists.

The global nature of these contributions is worth noting. The authors hail from a wide range of countries and continents, spanning North America, Europe, Asia, and beyond. This geographical diversity brings a rich tapestry of perspectives, research approaches, and cultural insights to the field of thyroid science. From bustling research centers in major metropolitan areas to institutions in smaller communities, these women represent the breadth of thyroid research worldwide. Their varied backgrounds not only showcase the international nature of scientific collaboration in this field but also underscore the importance of diverse voices in advancing our understanding of thyroid disorders and treatments. This Research Topic serves as a testament to the global reach and impact of women in thyroid science, demonstrating that excellence in this field has no geographical boundary. It also serves as a testament to the innovative and rigorous work being done by women researchers. By highlighting these contributions, we hope to inspire more young women to pursue careers in thyroid research and practice. Promoting gender diversity and inclusivity in our field will undoubtedly lead to new perspectives, ideas, and discoveries that will ultimately improve the lives of patients with thyroid disease.

We extend our sincere gratitude to all the authors who contributed to this Research Topic. Your work pushes the boundaries of thyroid science and provides an example for aspiring researchers. As we celebrate these achievements, let us also reflect on how we can continue to support and elevate women in thyroidology to ensure a bright future for our field.

